# GULP1/CED-6 ameliorates amyloid-β toxicity in a Drosophila model of Alzheimer’s disease

**DOI:** 10.18632/oncotarget.20062

**Published:** 2017-08-08

**Authors:** Wai Yin Vivien Chiu, Alex Chun Koon, Jacky Chi Ki Ngo, Ho Yin Edwin Chan, Kwok-Fai Lau

**Affiliations:** ^1^ School of Life Sciences, The Chinese University of Hong Kong, Shatin, NT, Hong Kong SAR

**Keywords:** CED-6, APP, Aβ, neurodegeneration, neurotoxicity, Gerotarget

## Abstract

Amyloidogenic processing of APP by β- and γ-secretases leads to the generation of amyloid-β peptide (Aβ), and the accumulation of Aβ in senile plaques is a hallmark of Alzheimer’s disease (AD). Understanding the mechanisms of APP processing is therefore paramount. Increasing evidence suggests that APP intracellular domain (AICD) interacting proteins influence APP processing. In this study, we characterized the overexpression of AICD interactor GULP1 in a *Drosophila* AD model expressing human BACE and APP695. Transgenic GULP1 significantly lowered the levels of both Aβ1-40 and Aβ1-42 without decreasing the BACE and APP695 levels. Overexpression of GULP1 also reduced APP/BACE-mediated retinal degeneration, rescued motor dysfunction and extended longevity of the flies. Our results indicate that GULP1 regulate APP processing and reduce neurotoxicity in a *Drosophila* AD model.

## INTRODUCTION

Human GULP1 (engulfment adaptor PTB-domain-containing 1), the homologue of *Caenorhabditis elegans* CED-6, is an adaptor protein with multiple protein interaction domains/regions including an N-terminal phosphotyrosine-binding (PTB) domain, a centrally located leucine zipper and a carboxyl terminal proline/serine rich region [1-3]. GULP1/CED-6 has been implicated in phagocytosis as it interacts with several engulfment receptors including CED-1, stabilin-1 and stabilin-2 [3-6]. Recently, we and others have shown that GULP1 PTB domain binds to Alzheimer’s disease amyloid precursor protein (APP) [7, 8].

APP is a large type I transmembrane protein contains a large ectodomain and a small intracellular domain, namely AICD. Amyloidogenic processing of APP by β- and γ-secretases leads to the generation of amyloid-β peptide (Aβ), and accumulation of Aβ to form senile plaques is a hallmark of Alzheimer’s disease (AD). Although the mechanisms by which APP processing is regulated are still not fully understood, increasing evidence suggests that AICD interacting proteins can influence APP metabolism and Aβ generation (see reviews [9, 10]). In fact, GULP1 is found to alter APP processing and Aβ generation in transfected cells [7, 8]. However, the effect of GULP1 on Aβ production *in vivo* remains to be determined.

To understand the effect of GULP1 *in vivo*, we utilized the fruit fly, *Drosophila melanogaster*. *Drosophila* is a popular model for studying neurodegenerative diseases, as approximately 75% of all known human disease-associated genes are conserved in flies, including those implicated in AD [11]. In addition, AD phenotypes such as learning disabilities and plaque deposition can also be observed in *Drosophila* [12, 13]. In this study, we established transgenic flies to overexpress GULP1 in an existing *Drosophila* AD model which expresses human APP695 wildtype and BACE genes. This *Drosophila* model is a powerful tool for AD research as the files exhibit a number of clinical AD neuropathology and symptomology for both familial/sporadic AD including Aβ aggregation and memory defects [14]. In this study, we observed that GULP1 reduces Aβ production, reduces retinal degeneration, rescues motor dysfunction and improves the life expectancy of the flies. Our data suggest that GULP1 is a potential modifier of neurotoxicity in AD by lowering Aβ levels.

## RESULTS

### Characterization of GULP1 transgene expression in *Drosophila*

**Figure 1:**
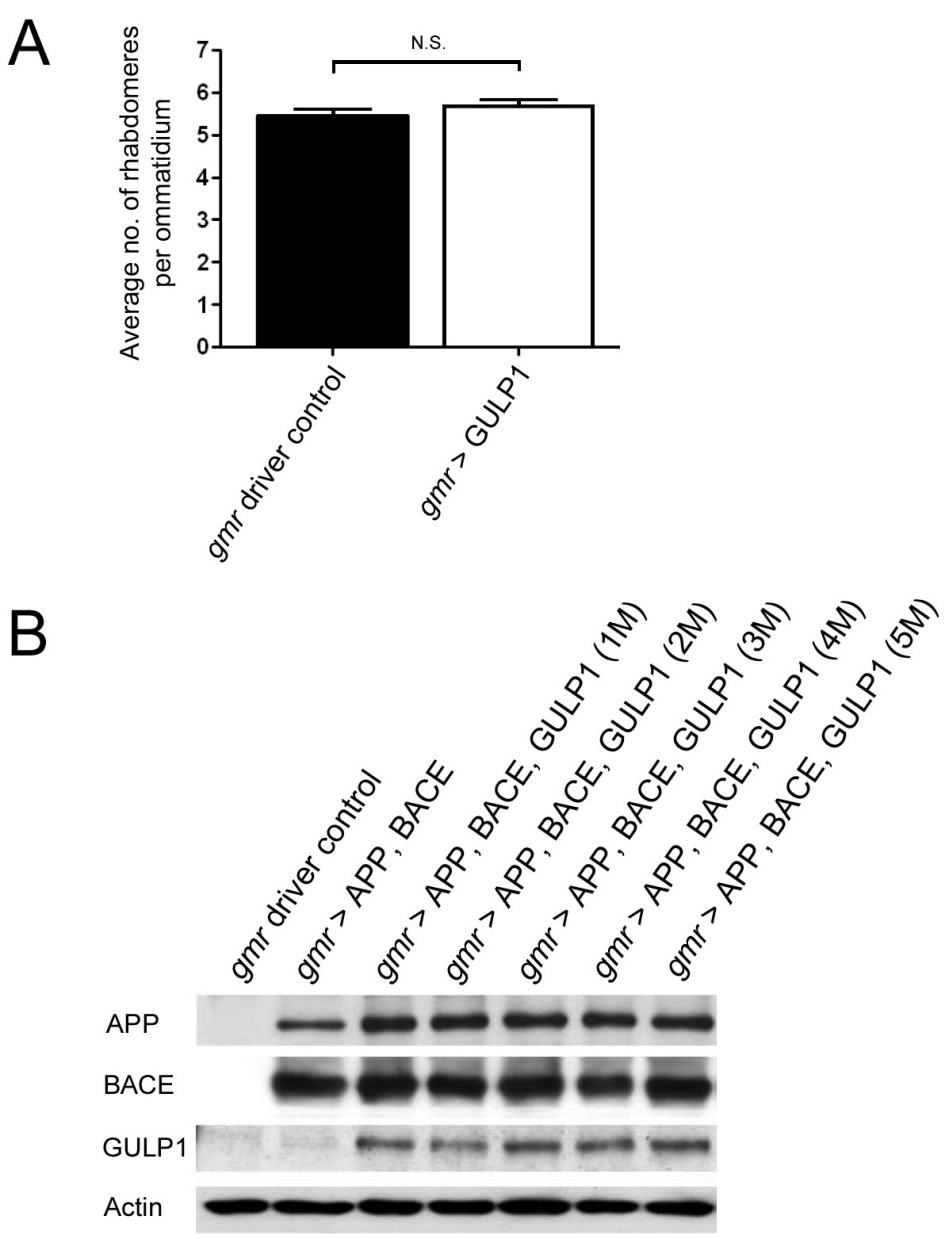
Characterization of GULP1 transgene in *Drosophila* **A.** Overexpression of GULP1 using *gmr-GAL4* does not change the overall retinal structure. Flies were from three independent experiments. The flies were raised at 19 °C and assayed at 5 dpe. **B.** Overexpression of human APP695, BACE and GULP1 proteins in *Drosophila.* Immunoblot analysis of fly head homogenates for APP, BACE, GULP1. 1M - 5M were independent transgenic flies of GULP1 under the UAS promoter, which were crossed into the fly disease model of APP and BACE expression driven by gmr-GAL4. Actin was used as loading control. Flies were raised at 28 °C and assayed at 6 dpe.

To determine the effect of GULP1 *in vivo*, we created *UAS-GULP1* transgenic flies, and crossed them to *gmr-GAL4* to overexpress GULP1 in the fly eyes. Expression of GULP1 in *gmr-GAL4*/+; *UAS-GULP1*/+ animals (*gmr > GULP1*) was confirmed by Western blot analysis (data not shown), and there was no significant change in the number of rhabdomeres per ommatidium between *gmr > GULP1* heterozygous and control *gmr* driver flies (Figure 1A). To investigate the effect of GULP1 on AD-like symptoms in flies, we utilized an existing *Drosophila* AD model which expresses human APP695 and BACE transgenes using *gmr-GAL4* (*gmr > APP, BACE*) [14]. We crossed the *UAS-GULP1* flies to the *gmr > APP, BACE* flies to produce *gmr-GAL4*/+; *UAS-APP695, UAS-BACE/UAS-GULP1* heterozygous flies (*gmr > GULP1, APP, BACE*). Western blot analysis revealed the expression of GULP1, APP and BACE transgenes in several independent lines (Figure 1B). There was no noticeable difference in GULP1 expression in different lines. The expression of a third transgene (GULP1) in the AD model did not result in a lower expression of APP or BACE due to the dilution of available GAL4. In contrary, the expression level of APP holoprotein was surprisingly increased by approximately 58% in *gmr > GULP1, APP, BACE* flies as compared to *gmr > APP; BACE* to flies (Figure 1B).

### Overexpression of GULP1 rescues motor dysfunction and extends life span in a *Drosophila* AD model

**Figure 2:**
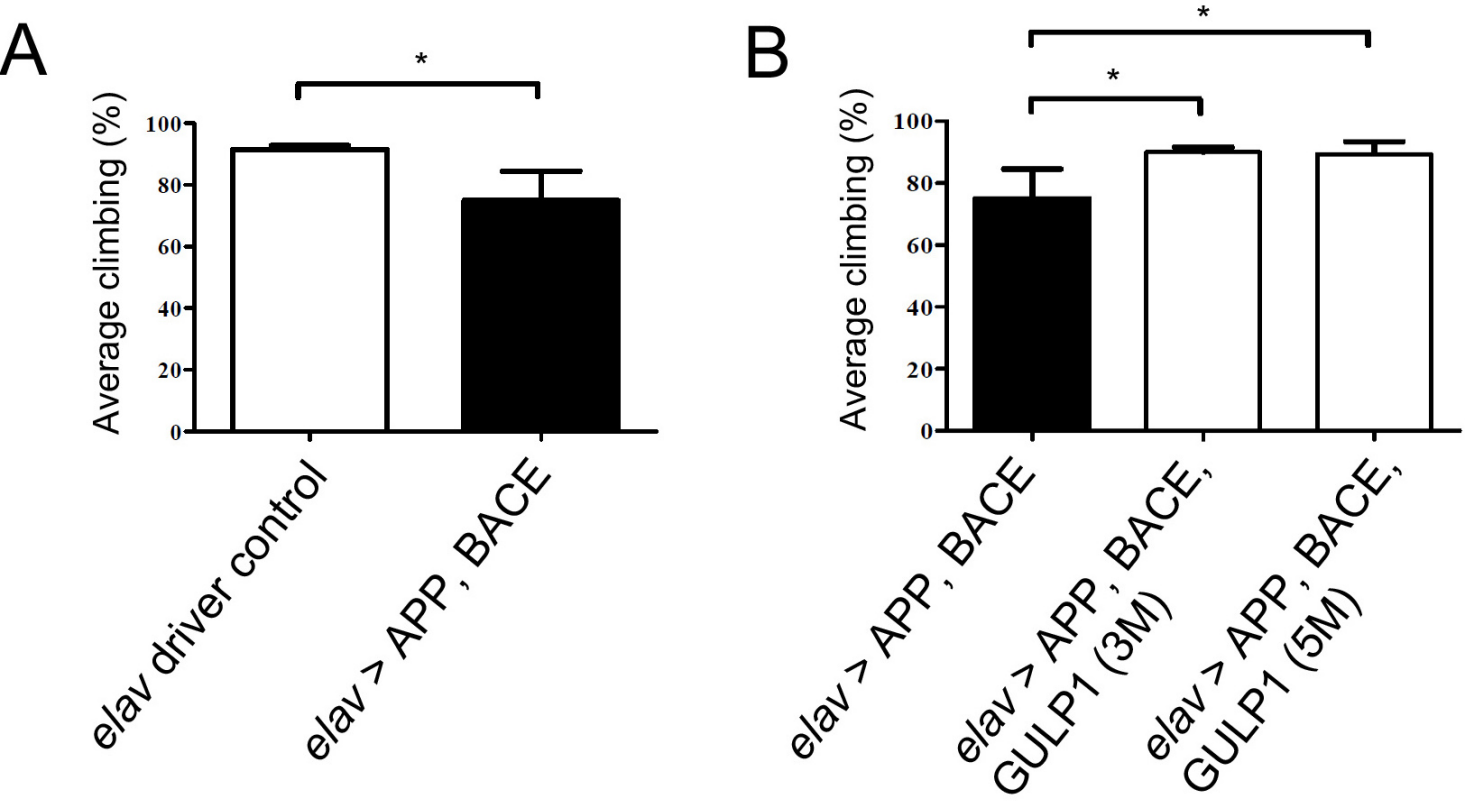
GULP1 ameliorates APP and BACE-induced locomotor dysfunction in a *Drosophila* AD model **A.** Compared with control *elav-GAL4* driver flies, overexpression of APP and BACE showed significant motor impairment; **B.** AD models expressing GULP1 delayed the impairment of locomotor function. The average climbing indices of *elav* driver control, *elav > APP, BACE, elav > APP, BACE, GULP1 (3M)* and *elav > APP, BACE, GULP1 (5M)* were 91%, 74%, 90% and 89% respectively. *N* = 80, *p < 0.05; error bars represent SD from at least 80 flies from three independent experiments. The flies were raised at 19 °C and assayed at 20 dpe.

Progressive decline in locomotor ability is one of the surrogate markers of neurotoxicity in a number of AD models [14-16]. To investigate if GULP1 can ameliorate APP/BACE-induced motor dysfunction, pan-neuronal *elav-GAL4* driver was used to overexpress two independent *UAS-GULP1* fly lines (3M and 5M) together with *UAS-APP695* and *UAS-BACE*. Similar to the previous report, we observed that *elav > APP, BACE* heterozygous flies have reduced climbing activities, indicating that APP and BACE compromised CNS functions [14] (Figure 2A). Remarkably, overexpression of GULP1 significantly improved the APP/BACE-induced motor impairment (Figure 2B).

**Figure 3:**
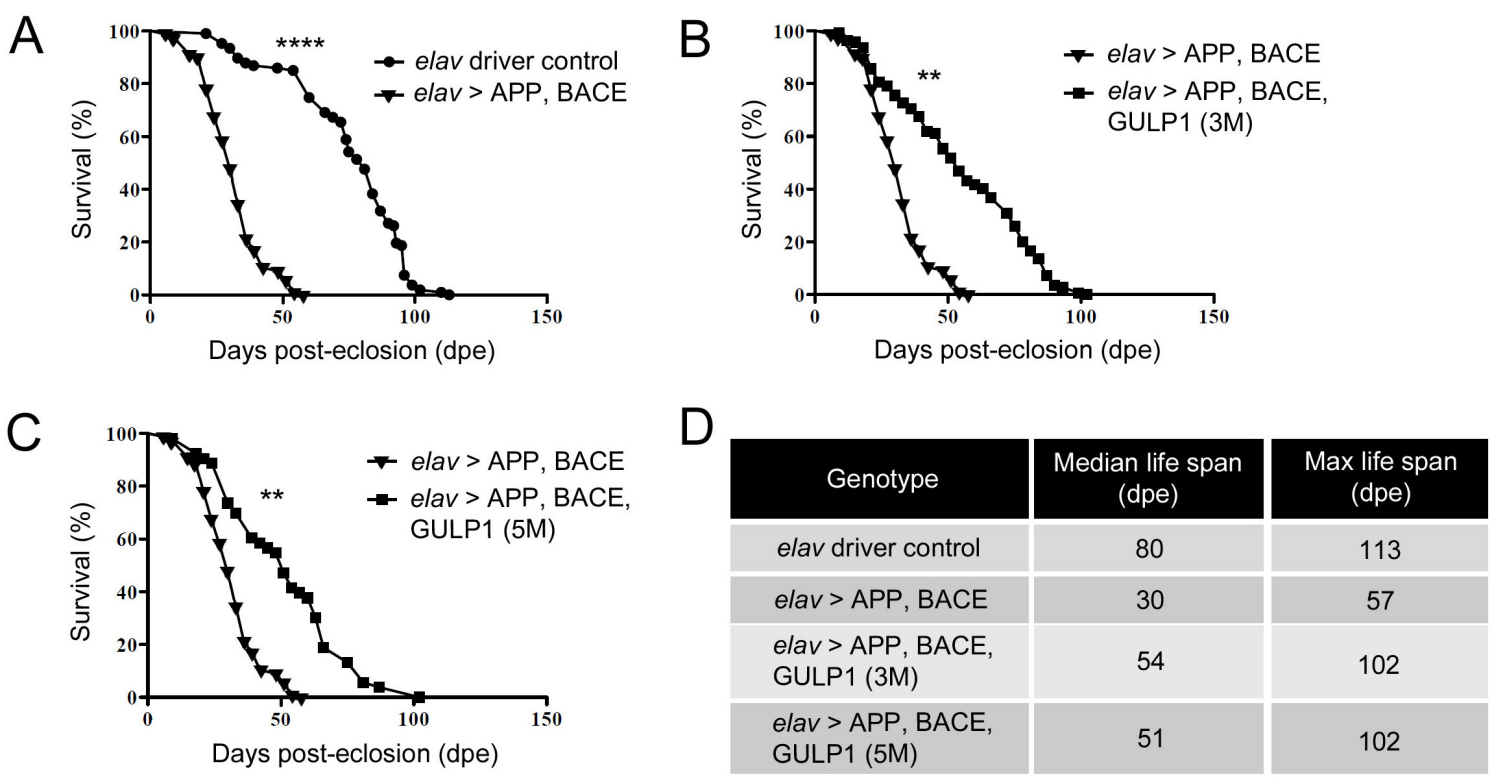
GULP1 extends life span in a *Drosophila* AD model **A.** Compared with control flies, overexpression of APP and BACE showed significantly shorter life span; **B.**-**C.** AD models expressing GULP1 demonstrated significantly longer survival time. **D.** Quantification of life span. *N* = 70. Log-rank (Mantel-Cox) test was performed. **** *p* < 0.0001, ** *p* = 0.0013, ** *p* = 0.0039 for **A.**, **B.** and **C.** respectively. At least 70 flies from three independent experiments were assayed. The flies were raised at 19 °C.

In addition, consistent with the previous report, the median survival time of *elav > APP, BACE* flies was markedly shorter than the heterozygous *elav-GAL4* control flies (30 days *vs* 80 days) (Figure 3A and 3D). On the other hand, overexpression of GULP1 increased the lifespan of the AD model to a median survival time to 54 days (Figure 3B-3D).

### GULP1 protects against APP/BACE-induced neurodegeneration in a *Drosophila* AD model

**Figure 4:**
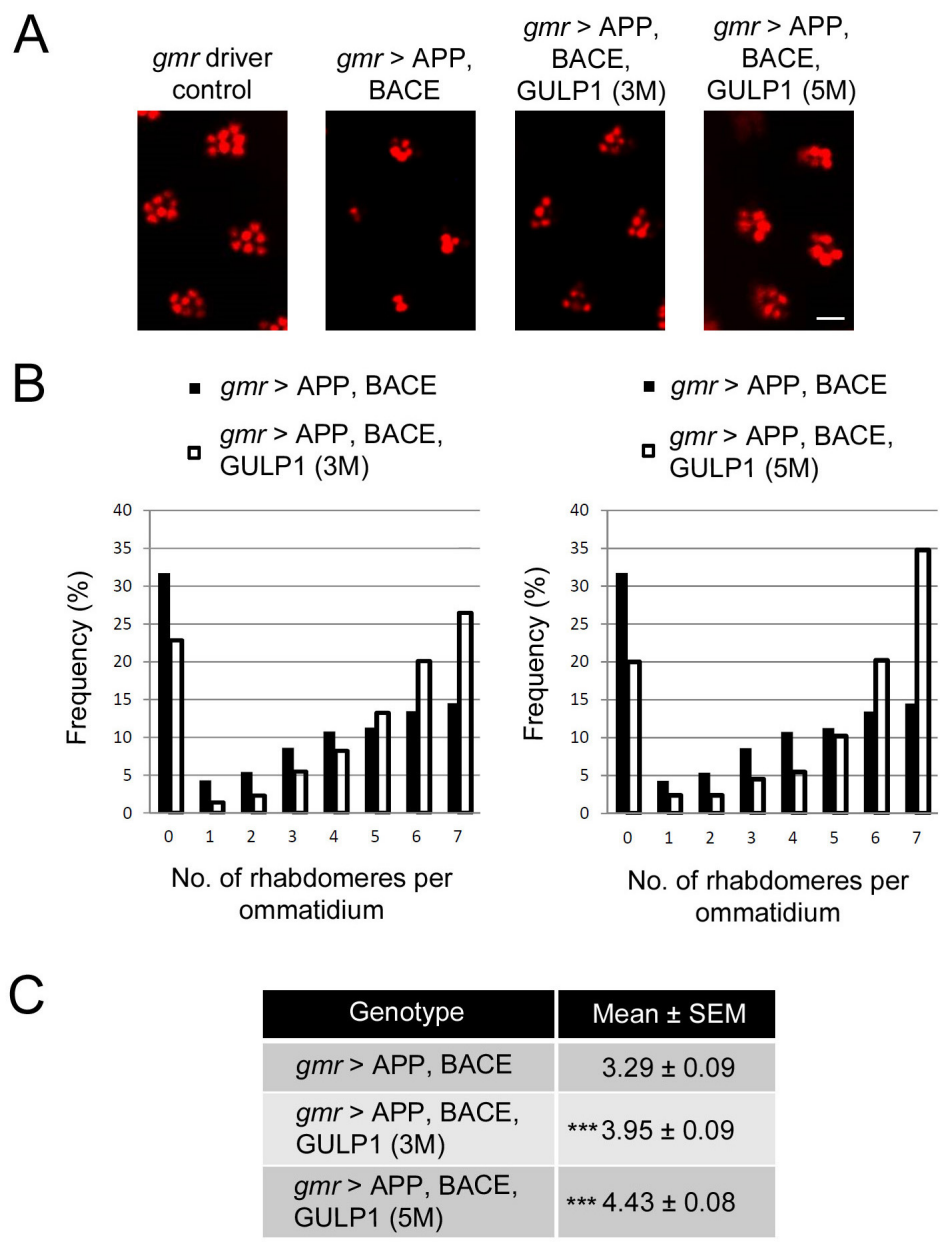
GULP1 reduces retinal degeneration in a *Drosophila* AD model **A.** Representative photomicrographs of fly retina in AD models with or without GULP1 expression from one of the three independent experiments. Scale bar is 7 µm. **B.** Distribution pattern of number of visible rhabdomeres per ommatidium in AD models with or without GULP1 expression from one experiment. Similar trend was observed in the other two independent experiments. **C.** Quantification of visible rhabdomeres. On average, 3.29 rhabdomeres per ommatidium were observed in flies expressing APP695 and BACE. Overexpression of GULP1 significantly suppressed the degeneration induced by APP and BACE expression, and increased the rhabdomere score to an average of 3.95 and 4.43 respectively. *N* = 300, ***p < 0.01. Flies were from three independent experiments. The flies were raised at 19 °C and assayed at 5 dpe.

The *Drosophila* compound eye has been widely used for monitoring neurotoxicity and neurodegeneration [17, 18]. To investigate the hypothesis that GULP1 reduces degeneration in the *Drosophila* AD model, we examined the ommatidial organization of *gmr > GULP1, APP, BACE* fly by pseudopupil assay. As shown in Figure 1B, overexpression of GULP1 did not show noticeable effect on the overall *Drosophila* retinal structure. Yet, GULP1 overexpression reduced the effect of APP/BACE-mediated degeneration as *gmr > GULP1, APP, BACE* flies showed significant higher average number of rhabdomeres per ommatidium than *gmr > APP, BACE* flies (Figure 4A-4C).

### Overexpression of GULP1 reduces Aβ generation in a *Drosophila* AD model

**Figure 5:**
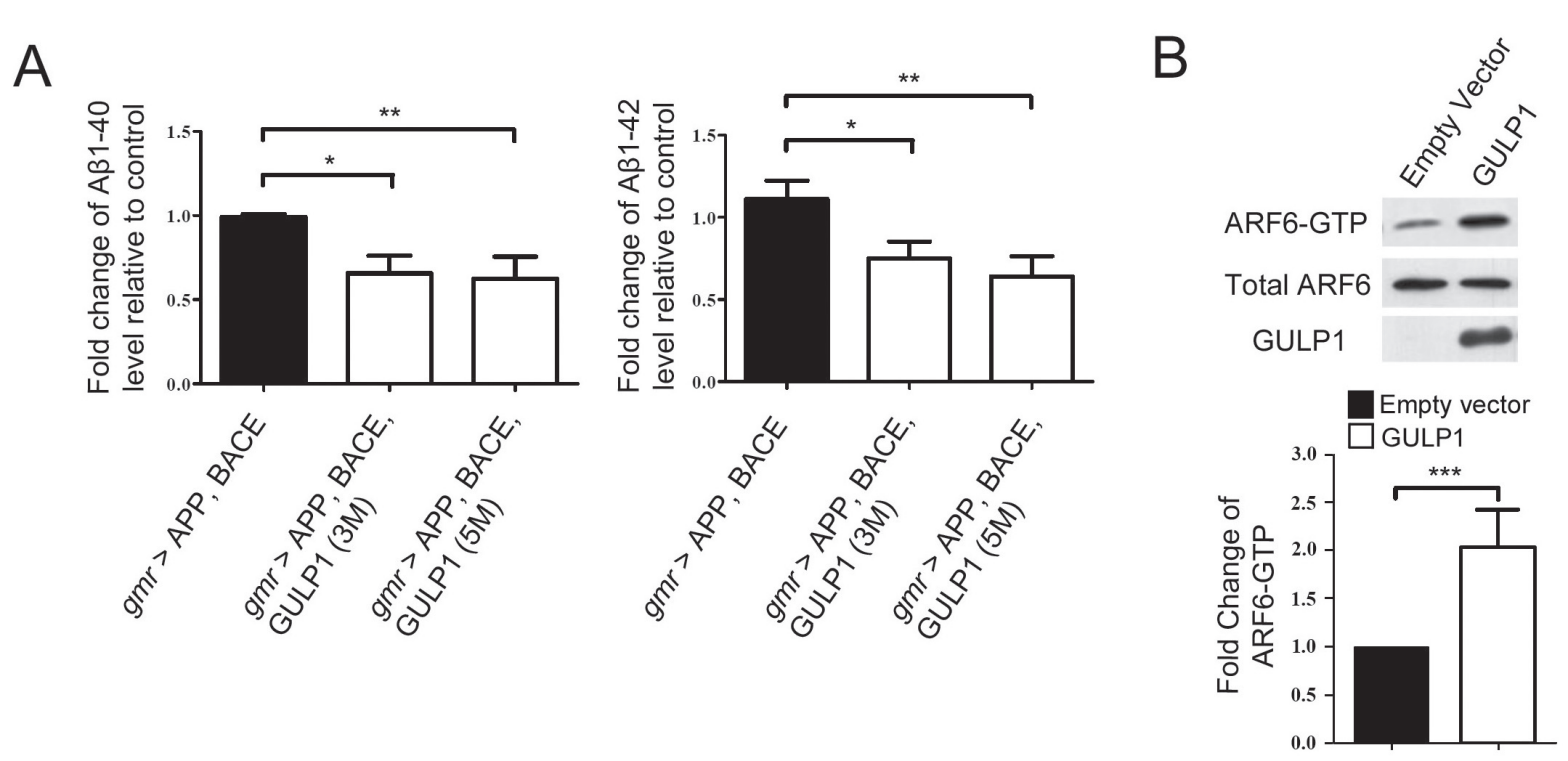
Overexpression of GULP1 decreases Aβ1-40 and Aβ1-42 levels in a *Drosophila* AD model **A.** Aβ1-40 and Aβ1-42 levels were measured by human Aβ ELISA. Both Aβ1-40 and Aβ1-42 decreased significantly in flies expressing GULP1. **p < 0.01, *p < 0.05; Error bars are SD from at least 120 flies from three independent experiments. The flies were raised at 28 °C and assayed at 6dpe. **B.** Total ARF6 levels and ARF6-GTP levels were examined in CHO cells overexpressing GULP1 by immunoblotting (Top panel). Bar chart shows relative ARF6-GTP amount. Data were obtained from three independent experiments. *N* = 3, *p < 0.001. Error bars are SD.

It is known that the overexpression of human APP and BACE increases amyloidogenic processing of APP and generation of Aβ in *Drosophila* [14, 19], and Aβ has been reported to shorten *Drosophila* lifespan and cause photoreceptor abnormality in the compound eye [20, 21]. Thus, it is possible that the mitigation of neurotoxicity by GULP1 is due to an overall reduction of Aβ production. To test this hypothesis, we overexpressed GULP1, APP and BACE using *gmr-GAL4* and examined GULP1’s effect on Aβ production. Indeed, we observed a significant reduction of Aβ1-40 and Aβ1-42 in *gmr > GULP1, APP, BACE* flies as compared to *gmr > APP, BACE* flies (Figure 5A), indicating that GULP1 rescues structural, behavioral and longevity phenotypes in the *Drosophila* AD model by lowering Aβ production levels.

Active ARF6 has been shown to lower Aβ generation by altering endosomal sorting of BACE1 in various mammalian cell types including primary neurons [22]. Moreover, GULP1 has been shown to alter the generation of APP C-terminal fragment β, a product of BACE1 cleavage of APP, in mammalian cells [7]. It is possible that GULP1 regulates APP processing *via* ARF6 in some fashion as the PTB domain of GULP1 interacts with ARF6, and the knockdown of GULP1 reduces ARF6 activation in mouse embryo fibroblast MEF 1 [23]. To test the hypothesis, we transfected cells with GULP1, and examined the levels of active ARF6 (ARF6-GTP). While the total ARF6 level did not change, more active ARF6 was observed in cells overexpressing GULP1 (Figure 5B). This suggests that GULP1 possibly reduces Aβ production and neurotoxicity *via* the activation of ARF6.

## DISCUSSION

Several lines of evidence from cell models suggest GULP1 modulates APP processing [7, 8]. However, the biological roles of GULP1 on Aβ productions *in vivo* and the subsequent physiological effects remain unknown. The current study provides first evidence that GULP1 affects human APP metabolism *in vivo* and improve structural, behavioral and longevity phenotypes in a *Drosophila* AD model.

The mechanism(s) by which GULP1 alters APP processing is largely unknown. GULP1 has been shown to interact with ARF6 [23], a member of the Ras superfamily of small GTPases functions in trafficking the membrane components between the plasma membrane and endosomal compartments (see review [24]). Several studies have shown that endosomes contain high level of BACE, and are a major subcellular compartment for Aβ production (see review [25]. In fact, active ARF6 has been shown to regulate endosomal sorting of BACE1 and lead to reduction in Aβ generation [22]. Here, we showed that overexpression of GULP1 could induce ARF6 activation. Hence, the effect of GULP1 on APP processing in *Drosophila* may be *via* activation of dARF6, the fly homolog of ARF6. Alternatively, dCED-6, the fly homologue of GULP1, has been reported to function as an *in vivo* clathrin adaptor for clathrin-mediated Yolkless uptake in *Drosophila* oocytes [26-28]. As APP internalization is also clathrin-mediated [29], it is possible that GULP1 regulates APP endocytosis processing in flies. It is also suggested that GULP1 may reduce maturation of APP along the secretory pathway and impair APP trafficking to the plasma membrane. Such retention of APP proteins in the secretory pathway consequently traps more of them inside the cells, limiting their processing by secretases at the plasma membrane, and ultimately reduced Aβ generation [8]. This is consistent with our observation that the level of APP appeared to be increased when GULP1 was overexpressed (Figure 1B).

In *Drosophila*, dCED-6 is a key molecule in the Draper pathway mediating the glial engulfment of dying/injured neurons as well as presynaptic debris at the larval neuromuscular junction [26, 30, 31]. Since *elav-GAL4*’s expression includes both neuronal and glial cells [32], it is possible that the overexpression of GULP1 in glia activated the Draper pathway, and facilitate the engulfment of extracellular Aβ. In fact, many previous studies have demonstrated the role of glial cells in Aβ clearance and degradation [33-38]. Additionally, the suppressive effect of GULP1 on Aβ level and reduced structural and behavioral abnormalities could be a result of potential neuroprotective effect of GULP1 on Aβ clearance as GULP1 is reported to stimulate the signaling of transforming growth factor-β (TGF-β) [39], a neurotrophic cytokine against Aβ toxicity [40].

On the other hand, conflicting effect of GULP1 on APP processing are reported [7, 8]. Similar controversies are reported for other AICD interacting proteins including FE65s and X11s [9, 41-49]. Noteworthy, GULP1, FE65s and X11s are adaptor proteins that functions in recruiting interactors for various biological pathways. One possible reason for such conflicting observations is that the cell types or models employed in the studies expressing different types and amounts of their interactors. Thus, in addition to ARF6, other GULP1 interactors may also participate in regulating APP processing. Moreover, a number of studies have revealed that the phosphorylation status of APP interactors would influence their effects on APP processing [50, 51]. Noteworthy, phosphoproteomic and mass spectrometric analyses from various laboratories have shown that GULP1 is a phosphoprotein [52-55]. Hence, the discrepancy in the effect of GULP1 on APP processing may also be a result of phosphorylation status of GULP1. Therefore, identification of the full spectrum of GULP1 interacting proteins and investigation of the role of GULP1 phosphorylation will provide further mechanistic insights into how GULP1 modulates APP processing.

Although lowering Aβ by the enhancement of Aβ clearance using recombinant Aβ antibodies have shown promises in mouse model [56], Eli Lilly recently announced that their Aβ antibody drug Solanezumab failed to demonstrate efficacy in an 18-month phase III clinical trial with over 2,100 participants. The exact reasons for the failure of the trial remain to be determined [57]. Solanezumab is thought to be function by sequestering Aβ to promote Aβ clearance. However, the drug could not lower Aβ generation. Therefore, combination therapy that increases Aβ clearance and suppresses Aβ production may be an alternative approach for AD. However, current strategies for reducing Aβ production remain unsatisfactory such as the use of γ-secretase inhibitors. Our finding that GULP1 reduces Aβ production in an AD *Drosophila* model does not only improve our understanding of the function of GULP1 in APP processing *in vivo*, but also opens a novel avenue for investigating the possibility of targeting GULP1-APP interaction to alter Aβ production.

## MATERIALS AND METHODS

### Generation of *UAS-GULP1* lines in *Drosophila*

The full length human GULP1 cDNA was amplified by PCR and subcloned into GAL4-responsive pUAST expression vector, and microinjected into *Drosophila* embryos (BestGene Inc, USA). The expressions of GULP1 in 5 independent lines (1M, 2M, 3M 4M and 5M) were determined by Western blotting.

### *Drosophila* stocks

Fly stocks and crosses were raised on standard cornmeal medium with 1.25% agar; 10.5% dextrose; 10.5% cornmeal and 2.1% yeast. Flies were kept in incubators (LMS Ltd., UK) maintained at 18 °C, 19°C, 25°C or 28°C as specified. *elav-GAL4* (458), *gmr-GAL4* (1104), *UAS-APP695, UAS-BACE* (33797) were obtained from Bloomington *Drosophila* Stock Center, USA. Virgin females for crosses were collected within 8 hours at 25°C or 16 hours at 18°C.

### Western blot analysis

Fifteen fly heads were collected and homogenized in 75 µl 2X SDS sample buffer containing 100mM Tris, 4% SDS, 0.2% Bromophenol blue, 20% Glycerol and 15 μl/ml β-mercaptoethanol. Lysates were then boiled for 10 minutes and separated by SDS/PAGE gels. Protein on the gels was transferred to nitrocellulose blotting membrane (PALL) using a wet blotting system (Bio-Rad). Blots were probed with the following antibodies: Anti-GULP1 [7]; Anti-APP [48]; Anti-BACE [49]; Anti-actin A2103 (Sigma).

### Aβ ELISA

Human Aβ1-40 and Aβ1-42 in the fly heads were determined by using the human β40 and β42 ELISA kits (Millipore). In brief, 15 fly heads were homogenized in 50 µl ice-cold 1X RIPA buffer containing 50 mM Tris, 150 mM NaCl, 1% SDS, 1% NP-40, 0.5% sodium deoxycholate, pH 8.0 and Complete™ protease inhibitor (Roche). The homogenates were diluted 10-fold with 450 µl sample diluent and then cleared by centrifugation at 15,000 rpm for 5 minutes at 4°C. 200 µl of supernatant of each sample was added to ELISA plate with primary antibody. After overnight incubation at 4°C, the ELISA plate was washed with wash buffer. Streptavidin-peroxidase-conjugate was then added for colorimetric signal development at room temperature. The colorimetric reaction was stopped by adding stop solution, and signals were measured at 450 nm by using a microplate reader (Bio-Rad).

### Pseudopupil assay

Pseudopupil assay was performed essentially as described previously [58, 59]. In brief, 5 days post-eclosion (dpe) fly eyes were examined under a light microscope (Olympus CX31) with a 60X oil objective. At least 200 ommatidia from at least 15 adult flies obtained from three independent crosses were used to calculate the average number of rhabdomeres per ommatidium.

### Locomotor activity assay

Locomotor activity of the flies were determined at 20 dpe. In brief, group of 10 flies were placed at the bottom of a 15mL falcon tube. The number of flies that successfully climbed up a vertical distance of 8 cm or more was recorded. At least 80 flies of each genotype were analyzed in an experiment. Three independent experiment were performed.

### Longevity assay

Groups of 15 flies were placed in a food vial. Dead flies were counted every 3 days. At least 70 flies were assayed for each genotype from three independent crosses.

### ARF6 activation assay

ARF6 activation assay was performed as described previously [60] by an active ARF6 pull-down kit (Cell Biolabs). ARF6 was detected by a mouse anti-ARF6 supplied from the kit.

### Statistical analysis

The Mann-Whitney rank sum test was performed to compare mean differences between the average numbers of rhabdomeres per ommatidium in pseudopupil assay. One-way ANOVA test with Tukey post-hoc analysis was performed for ELISA analysis and climbing assay. Log-rank (Mantel-Cox) Test was performed for survival assay. A P-value of less than 0.05 was considered statistically significant. Significance is indicated as *p < 0.05, **p < 0.01.
